# Phosphatidylinositol 4,5-bisphosphate in the Control of Membrane Trafficking

**DOI:** 10.7150/ijbs.49665

**Published:** 2020-08-25

**Authors:** Suhua Li, Chinmoy Ghosh, Yanli Xing, Yue Sun

**Affiliations:** 1 Philips Institute for Oral Health Research, School of Dentistry and Massey Cancer Center, Virginia Commonwealth University, Richmond, VA 23298, USA; 2 Department of Otolaryngology, Shanghai Pudong New Area Gongli Hospital, Shanghai, China

**Keywords:** PI4, 5P_2_, membrane trafficking, PIPK, endosome, lysosome, autophagy

## Abstract

Phosphoinositides are membrane lipids generated by phosphorylation on the inositol head group of phosphatidylinositol. By specifically distributed to distinct subcellular membrane locations, different phosphoinositide species play diverse roles in modulating membrane trafficking. Among the seven known phosphoinositide species, phosphatidylinositol 4,5-bisphosphate (PI4,5P_2_) is the one species most abundant at the plasma membrane. Thus, the PI4,5P_2_ function in membrane trafficking is first identified in controlling plasma membrane dynamic-related events including endocytosis and exocytosis. However, recent studies indicate that PI4,5P_2_ is also critical in many other membrane trafficking events such as endosomal trafficking, hydrolases sorting to lysosomes, autophagy initiation, and autophagic lysosome reformation. These findings suggest that the role of PI4,5P_2_ in membrane trafficking is far beyond just plasma membrane. This review will provide a concise synopsis of how PI4,5P_2_ functions in multiple membrane trafficking events. PI4,5P_2_, the enzymes responsible for PI4,5P_2_ production at specific subcellular locations, and distinct PI4,5P_2_ effector proteins compose a regulation network to control the specific membrane trafficking events.

## Introduction

Membrane trafficking, the process using membrane-bound vesicles as transport intermediaries, controls the flow of proteins and other macromolecules materials between different subcellular compartments 1, 2. This process is also critical for the communication between the cell and its environment. Normal membrane trafficking processes are essential for maintaining cellular homeostasis and is critical for regulating fundamental processes such as cell signaling, nutrient uptake, immune responses, membrane turnover, and development 3-5.

**Figure 1:**
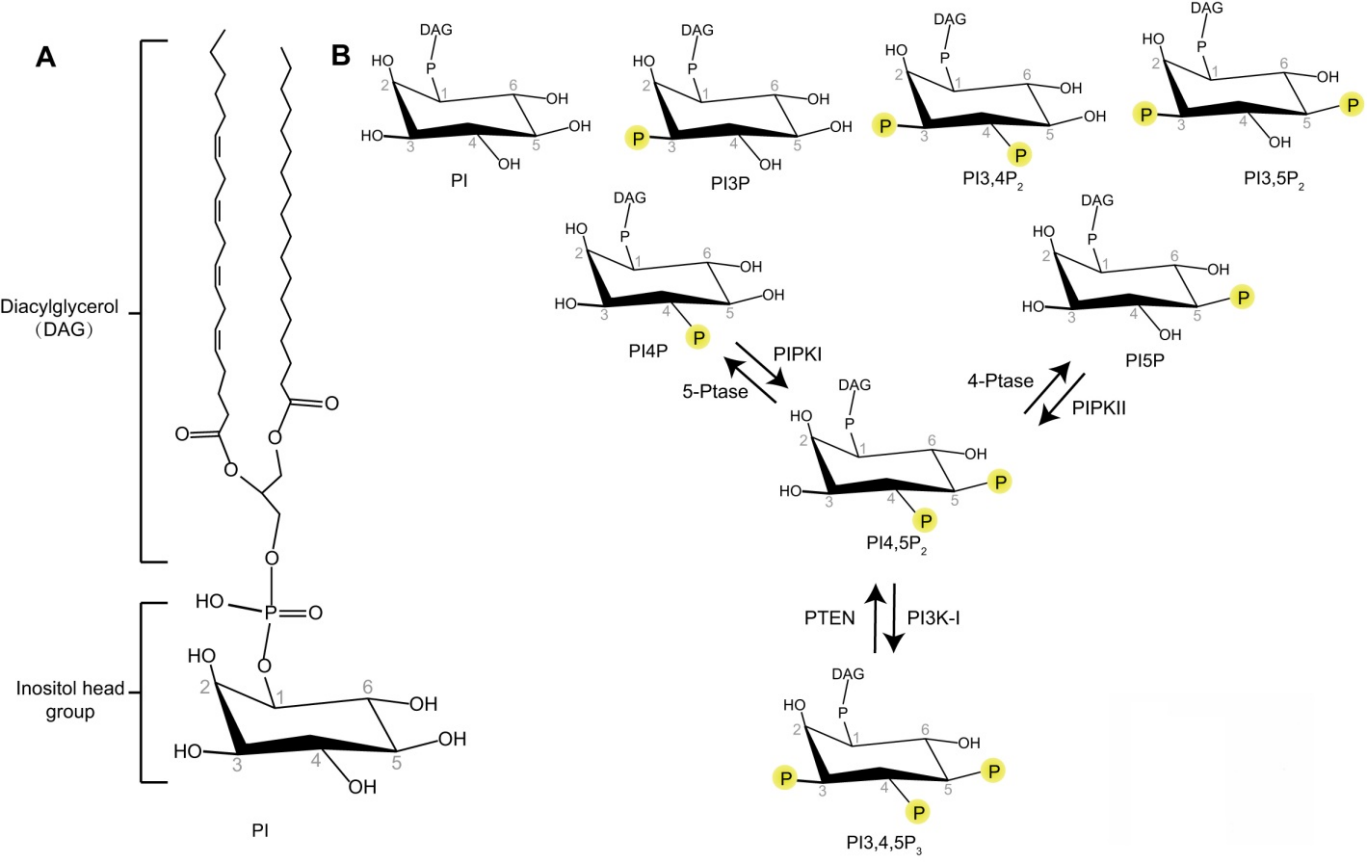
** Schematic representation of the phosphoinositides. (A)** Phosphatidylinositol (PI) contains an inositol head group connected to a Diacylglycerol (DAG) via a phosphodiester linkage. (B) Phosphate groups can be attached to any permutation of 3-, 4- and 5-hydroxyl groups of the inositol head of PI to produce different phosphoinositides. The interconversion of PI4,5P_2_ with PI4P, PI5P, or PI3,4,5P_3_ by phosphorylation and dephosphorylation are indicated.

Phosphoinositides are membrane-containing phospholipids that play a central role in the modulation of membrane trafficking by controlling the membrane dynamics and vesicular transport. They are differentially phosphorylated at the 3, 4, or 5 positions of their inositol ring. Seven phosphoinositide species have been identified as phosphatidylinositol 3-phosphate (PI3P), phosphatidylinositol 4-phosphate (PI4P), phosphatidylinositol 5-phosphate (PI5P), phosphatidylinositol 3,5-bisphosphate (PI3,5P_2_), phosphatidylinositol 4,5-bisphosphate (PI4,5P_2_), phosphatidylinositol 3,4-bisphosphate (PI3,4P_2_), and phosphatidylinositol 3,4,5-trisphosphate (PI3,4,5P_3_) (**Figure 1**) 6. Different phosphoinositide species can be rapidly converted to each other by phosphorylation or dephosphorylation via specific lipid kinases or phosphatases. The unique distribution of different phosphoinositides species at specific intracellular membranes makes these molecules uniquely suited to direct specific membrane trafficking events 7. Some studies indicate that PI3P is accumulated at early endosomes, PI4P at the membrane of Golgi apparatus, PI3,5P_2_ at late endocytic compartments, and PI4,5P_2_ at the plasma membrane 7. PI3P regulates membrane dynamics at early endosomes, phagosomes, and autophagosomes 7. PI3,5P_2_ controls late endosomal dynamics 8, 9. PI4P is a central regulator of Golgi function 7. As PI4,5P_2_ is enriched at the plasma membrane, the research of PI4,5P_2_ function in membrane trafficking is first focused on membrane dynamics at the plasma membrane such as endocytosis and exocytosis 10, 11. However, recent research works reveal the important roles of PI4,5P_2_ in many other essential membrane trafficking events, such as endosome maturation, trafficking between endosome and Golgi, lysosomal sorting, and autophagy 12-15. This review will provide a snapshot of how PI4,5P_2_ pathway controls different membrane trafficking events.

## Kinases producing PI4,5P_2_

PI4,5P_2_ is produced by type I and type II phosphatidylinositol-phosphate kinases (PIPKs) (**Figure 1**) 16. Type I PIPKs (PIPKIs) subfamily members preferentially utilize PI4P as the substrate to generate the majority of PI4,5P_2_ in the cell. Type II PIPKs (PIPKIIs) prefer to synthesize PI4,5P_2_ using PI5P as substrate. The cellular PI4P concentration is much higher (at least 20-fold) than those of PI5P 6, 17. Therefore, the majority of cellular PI4,5P_2_ is produced by PIPKIs by phosphorylating PI4P 18. In addition, a small amount of PI4,5P_2_ can also been produced by dephosphorylation of PI3,4,5P_3_ via the phosphatase and tensin homolog (PTEN) (**Figure 1**) 19.

Both of the PIPKIs and PIPKIIs subfamilies have three isoforms α, β and γ 18. Distinct genes encode each different isoforms. Furthermore, the PIPK genes undergo alternative splicing, which generate multiple splice variants and further confers the diversity of PIPKs 20, 21. Different PIPK isoforms present unique tissue and subcellular distribution patterns. For example, PIPKIα is mainly found in membrane ruffles and the nucleus, whereas, PIPKIβ is found to majorly distribute to the perinuclear region 18. The subcellular distribution of PIPKIγ isoforms is highly diverse including cell membrane, focal adhesions, and endosomes, which is due to the existence of multiple PIPKIγ alternative splicing variants. In human, at least six PIPKIγ splice variants have been identified and named as PIPKIγi1-i6 22, 23. These splice variants can play distinct function in specific membrane trafficking events. For example, PIPKIγi2 is found at cell adhesions and can mediate the sorting of β1 integrin-containing secretory vesicles to plasma membrane by interacting with the exocyst complex 24. PIPKIγi5 is located to endosomes and is required for Epidermal Growth Factor Receptor (EGFR) sorting from endosome to lysosome for degradation 12. These different PIPKI isoforms and splice variants contain a similar kinase core domain with conserved catalytic residues that bind ATP and Mg^2+^ and the residues to recognize the specific lipid substrate 16. Outside the kinase core region, the diversity of PIPKI sequences mediate their interaction with specific targeting proteins, which then recruit these kinases to distinct sub-cellular locations to produce PI4,5P_2_. Remarkably, many of these targeting proteins are also PI4,5P_2_ effectors, which means they can bind with PI4,5P_2_ and their function is modulated by PI4,5P_2_.

**Figure 2:**
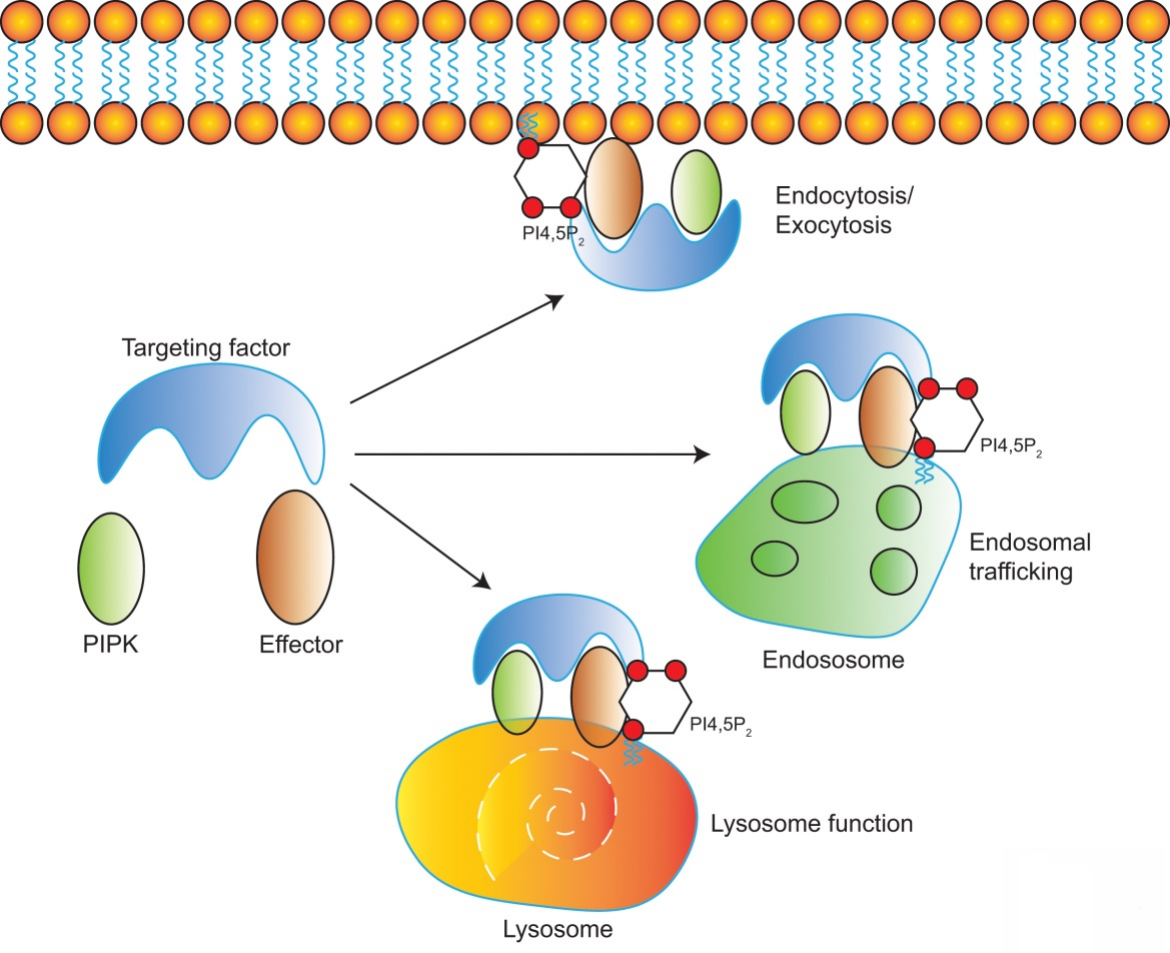
** Model of PI4,5P_2_, PIPKs, and PI4,5P_2_-effectors in the regulation of multiple membrane trafficking events.** PIPKs can be recruited by different targeting factors to distinct subcellular locations such as plasma membrane, endosomes, and lysosomes. Then the local production of PI4,5P_2_ by PIPKs recruits specific PI4,5P_2_-effectors to modulate different membrane trafficking events.

Diverse PIPKs, specific PI4,5P_2_ effectors, and distinct subcellular PI4,5P_2_ pools compose a complicate signaling network to control different membrane trafficking events such as clathrin-mediated endocytosis, exocytosis, endosomal trafficking, hydrolases sorting to lysosomes, and autophagy (**Figure 2**). PIPKs play a central role in the spatial and temporal control of PI4,5P_2_ subcellular production and function. Different PIPKs can be recruited to distinct subcellular locations and produce PI4,5P_2_ in local. Then the subtle change of local PI4,5P_2_ levels modulates the function of specific PI4,5P_2_ effectors to induce diverse biological effects to control specific membrane trafficking events.

## Endocytosis

**Figure 3:**
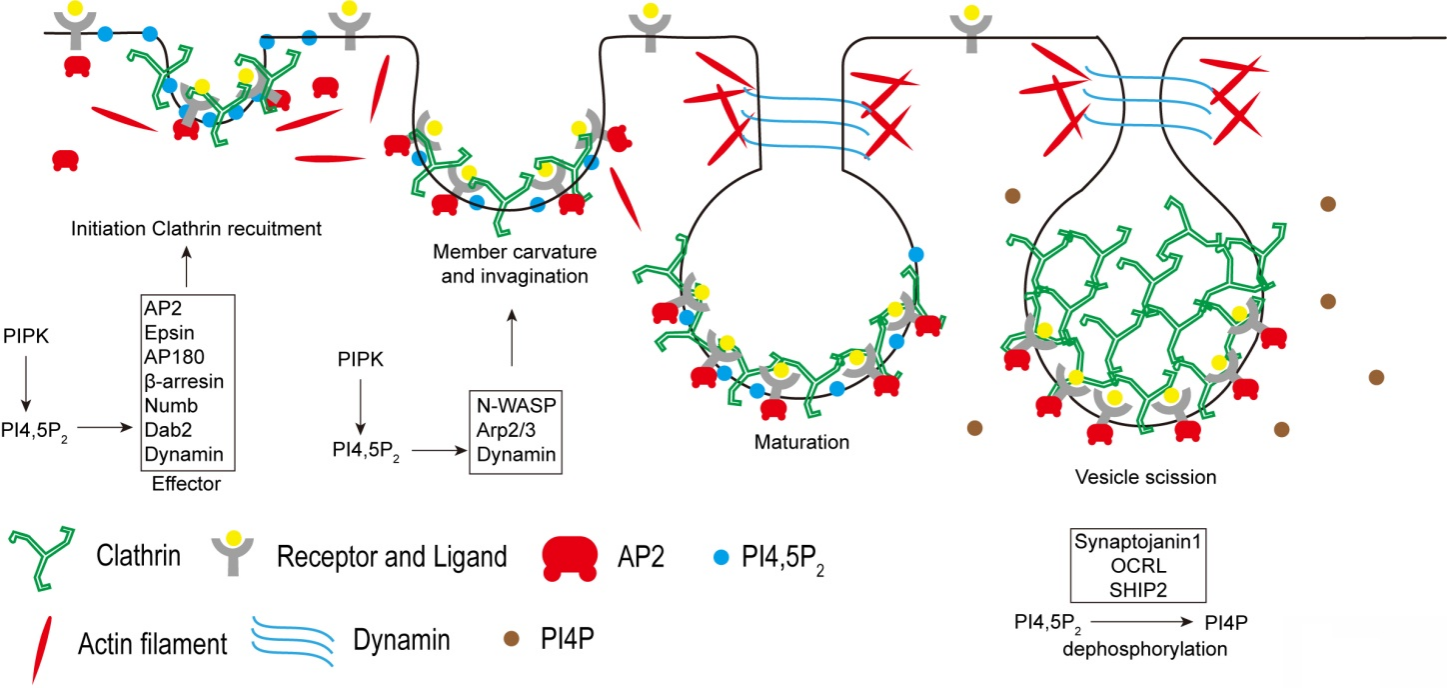
** PI4,5P_2_ in the regulation of Clathrin-mediated endocytosis.** PI4,5P_2_ recruits many clathrin adaptors such as AP2, Epsin, AP180, β-arrestin, Numb, Dab2, and Dynamin to plasma membrane and modulates their functions, which is required for clathrin recruitment to membrane to initiate the formation of clathrin-coated pits. Then by modulating N-WASP, Arp2/3, and Dynamin, PI4,5P_2_ is required for induce membrane curvature, invagination, and maturation of clathrin-coated vesicles. After final scission step, clathrin need to be released from the internalized vesicles. The down-regulation of PI4,5P_2_ by phosphoinositide phosphatases synaptojanin 1, OCRL, and SHIP2 at the clathrin-coated vesicles is required for the clathrin releasing.

Endocytosis is a type of active transport that engulfs a wide range of cargo molecules from the cell surface to the interior. This process is essential for cells to the control of signal transduction, to the uptake of nutrients, and to the regulation of plasma membrane composition in response to environmental changes. Although multiple endocytic pathways have been identified in eukaryotic cells, the best characterized pathway is clathrin-mediated endocytosis (CME) 25. It is the major endocytic route in mammalian cells and responsible for most of the uptake of transmembrane receptors and transporters to regulate cell surface signaling 26, 27. CME is a multistage process: nucleation of clathrin-coated pits; cargo capture in coated pits; clathrin coat growth to induce curvature and membrane invagination to form a pocket around the target particle; vesicle scission and clathrin uncoating to finally release the particle vesicle to the interior of the cell 28. PI4,5P_2_ pathway plays critical roles in the modulation of CME (**Figure 3**).

Being the most abundant phosphoinositide species found at plasma membrane, PI4,5P_2_ plays critical role in the control of CME 29. PI4,5P_2_ production is required for the recruitment of clathrin to plasma membrane by modulating the function of clathrin adaptor proteins. Clathrin does not directly bind to membranes. Therefore, the adaptors are required to recruit clathrin and to stabilize its association with the membrane. Many clathrin adaptors, such as the AP2 complex, Epsin, AP180, dynamin, β-arrestin, Numb, and Dab2 are PI4,5P_2_ effectors 30-33. AP2 complex is one of the key clathrin adaptors composed of two large subunits, α- and β2-adaptin and two smaller subunits, μ2- and σ2-adaptin 34. PI4,5P_2_ initially binds with the AP2 α-adaptin subunit, which leads to a conformational change in the μ2 subunit to expose the binding sites for PI4,5P_2_ and the cargo sorting YXXΦ motifs. Thus, PI4,5P_2_ strongly enhances the AP2 association with plasma membrane 35. In turn, AP2 recruits clathrin to the membrane to initiate the formation of clathrin-coated pits. PI4,5P_2_ also binds with other AP2 adaptors including epsin, AP180, Dab2, and β-arresin to further promote the clathrin recruitment to plasma membrane 36. Besides the roles in clathrin recruitment, PI4,5P_2_ also coordinates with the ENTH and BAR domains of membrane proteins to regulate membrane deformation to assist in the formation of vesicles 32, 37, 38. The membrane deformation also requires the coordination of actin cytoskeleton to generate force. PI4,5P_2_ can modulate actin cytoskeleton rearrangement via the actin regulating proteins, including N-WASP and Arp2/3 complex 39, 40. PI4,5P_2_ activates N-WASP by binding to a short polybasic region to release N-WASP auto-inhibition. Therefore, by recruiting and activating N-WASP and Arp2/3 complex, PI4,5P_2_ stimulates actin polymerization, which is important for membrane deformation during CME 41. PI4,5P_2_ is also required for the vesicle scission process at the final stages of CME. The GTPase dynamin, a scission factor, can be recruited and activated by PI4,5P_2_ at the membrane necks, which is required to force generating events to release the endocytic vesicle from the plasma membrane 29, 42.

PIPKIs are the major enzymes responsible for PI4,5P_2_ synthesis at plasma membrane. AP2 directly interacts with PIPKI via its µ2 subunit 43. The cargo protein binding with µ2 can further stimulate PIPKI activity to enhance PI4,5P_2_ production at the CME sites 43. Knockdown of PIPKIβ or PIPKIγ isoform inhibits transferrin receptor internalization via CME 30, 44, which supports the function of PIPKI in controlling CME. At the synapse, PIPKIγ is highly expressed and is critical for maintaining the PI4,5P_2_ levels 45, 46. Knockout of PIPKIγ in mouse dramatically decreases the PI4,5P_2_ levels at synapse 45, 46. On the contrary, the poly-phosphoinositide phosphatase synaptojanin 1 dephosphorylates PI4,5P_2_ to down-regulate it levels at synapse 47. The disruption of synaptojanin 1 enhances PI4,5P_2_ levels and causes an increase of clathrin-coated intermediates accumulated at the endocytic zones 46, 48. PIPKIγ and synaptojanin 1 antagonize each other in the regulation of PI4,5P_2_ levels and in the recruitment of clathrin coats to plasma membranes 46. Including the mammalian systems, the function of PI4,5P_2_ and PIPKs in endocytosis is also reported in many other systems such as in plant and yeast. Phosphatidylinositol-4-phosphate 5-kinase 6 (PIP5K6) is a pollen-enriched *Arabidopsis* PIPKI that is mainly located to the subapical plasma membrane in pollen tubes 49. Knockdown of PIP5K6 disrupts the assembly of clathrin onto the apical plasma membrane and impairs endocytosis in pollen tubes 49. In yeast, Mss4 is the only one kind of PIPKI and it is essential for PI4,5P_2_ production and endocytosis 6, 50-52.

At the final step of CME, once the coated vesicles are pinched off from the plasma membrane, the uncoating of clathrin is required for the vesicles to enter subsequent stages of the endocytic trafficking pathway. The recruitment of Rab5 to coated vesicles disrupts AP2-PI4,5P_2_ binding 53. Then the poly-phosphoinositide phosphatase synaptojanin 1 down-regulates PI4,5P_2_ levels by converting PI4,5P_2_ to PI4P. Subsequently, auxillin and Hsc70 collaborate to disassemble the clathrin coat 54. Knockout of Synaptojanin 1 leads to accumulation of clathrin-coated vesicles and delays the vesicle re-availability 55. Other PI4,5P_2_ 5-phosphatases such as oculocerebrorenal syndrome of Lowe protein (OCRL) and Src-homology 2 containing 5-phosphatase 2 (SHIP2) can also be recruited to clathrin-coated vesicles to decrease PI4,5P_2_ levels. Loss of these phosphatases impairs CME and causes accumulation of clathrin-coated vesicles 56-58. Thus, the down-regulation of PI4,5P_2_ levels at the clathrin-coated vesicles is a key step for the clathrin uncoating and is required for the recycle of clathrin to form newly coated vesicles.

PIPKIs use PI4P as immediate precursor to make PI4,5P_2_ by adding phosphorylation at the 5-position of PI4P inositol ring. There are at least two supplying pools of PI4P to support the PI4,5P_2_ levels at plasma membrane: one is the PI4P pool directly produced at the plasma membrane, the other is from the Golgi 59. Specific depletion of PI4P at plasma membrane or Golgi only causes small amount of PI4,5P_2_ levels decreasing 59-62. While depleting PI4P simultaneously at both plasma membrane and Golgi leads to a larger amount decrease of PI4,5P_2_ levels at the plasma membrane 59.

Except the CME, there are also some clathrin-independent endocytosis (CIE) pathways are identified including Caveolae-dependent, RhoA-regulated, ARF6-regulated, and CDC42-regulated CIE pathways 63. The molecular mechanisms controlling CIE are still in the beginning of recognition. Although the reported research works of PI4,5P_2_ function in CIE are much less compared with the works in CME, some reports suggest that PI4,5P_2_ may also play important roles in the regulation of CIE. For example, PI4,5P_2_ and its producing kinase PIPKI have been found to modulate ARF6-regulated CIE. Small GTPase Rac1 binding with PIPKI can induce PI4,5P_2_ production at ARF6-positive plasma membrane invagination tubules, which is required for ARF6-regulated CIE 64, 65. Cortactin and dynamin are PI4,5P_2_-binding proteins and they play critical roles in CIE 66, 67. PI4,5P_2_ may regulate their function in CIE by modulating cortactin- or dynamin-dependent actin assembly and membrane-remodeling. More research are required to further understand the detail function of PI4,5P_2_ in CIE.

## Endosomal trafficking and endosome maturation

Endosomes are major protein-sorting stations inside the cells 68. Once entering the cell, the endocytic vesicles containing cargoes internalized from the plasma membrane will go through multiple rounds of homotypic fusion to form early endosomes 69, 70. Cargoes from the Golgi apparatus can also be transported to endosomes 71. Then the fate of these cargoes is determined by the subsequent endosomal sorting process. From endosomes, these cargoes are sorted to lysosomes for degradation or conversely sorted for retrieval and subsequent recycling to various membrane compartments 68. In this way, endosomes controls the trafficking and turnover of many important proteins including signaling receptors, lysosomal hydrolase receptors, nutrient transporters, and adhesion molecules 68. Defects in endosomal sorting are implicated in various human pathologies, such as cancer and neurodegenerative disorders 72-74. Researches of phosphoinositides function in the control of the endosomal system are first focused on PI3P and PI3,5P_2_ for these two phosphoinositide species are most abundant at the endosomes 75. The important roles of PI4,5P_2_ in this system has only recently been recognized 12, 13, 76.

**Figure 4:**
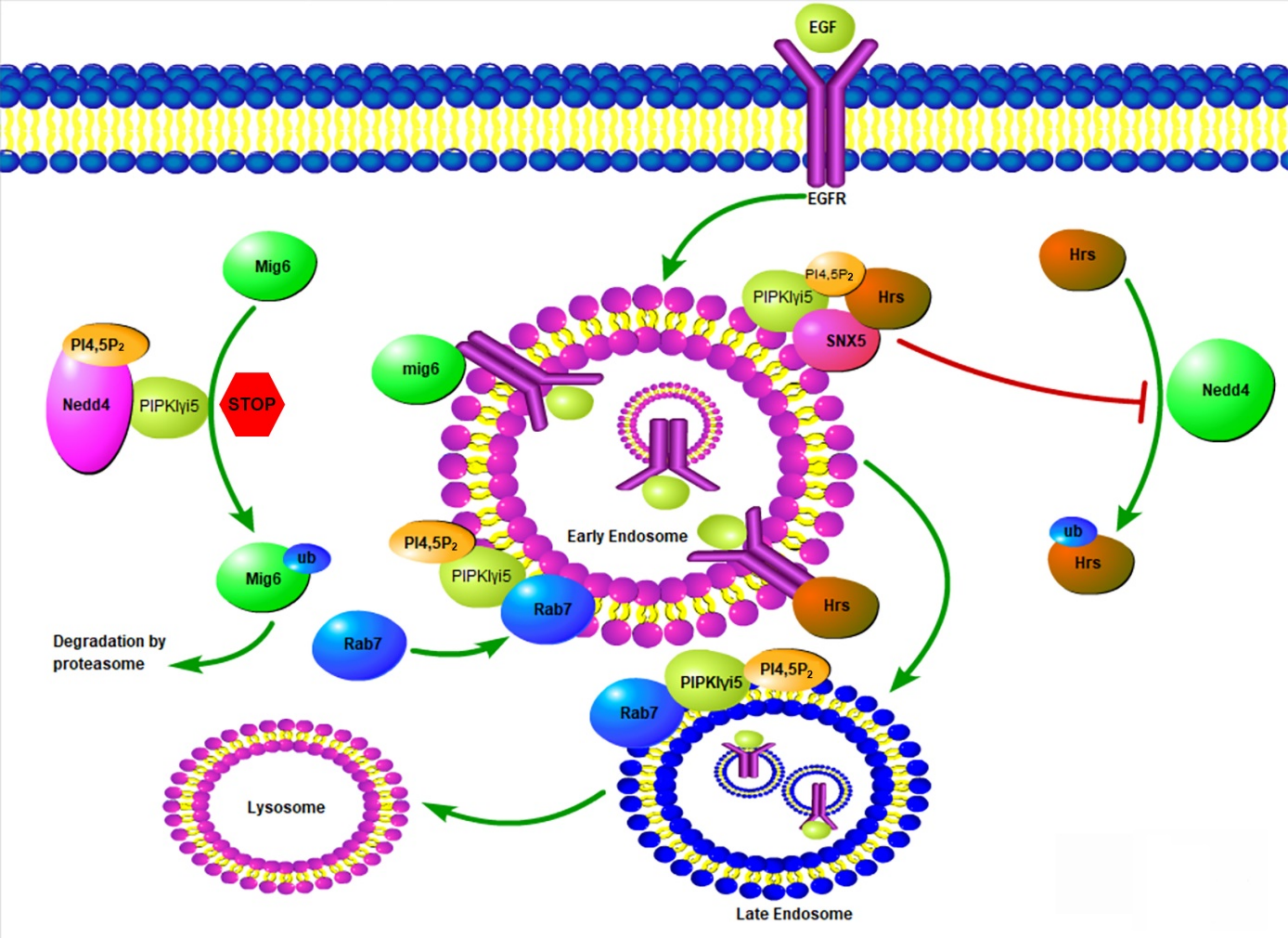
** Model of PIPKIγi5 and PI4,5P_2_ in the modulation of EGFR endosomal trafficking.** PIPKIγi5, an enzyme producing PI4,5P_2_, controls the endosomal sorting of EGFR by modulating Hrs-EGFR interaction, Mig6 degradation, and Rab7 recruitment to early endosomes. Hrs can recognize ubiquitinated EGFR at the limiting membrane of early endosomes, and then recruit other component of ESCRT complex to initiate the membrane invagination and EGFR sorting into the intraluminal vesicles (ILVs). Hrs can be ubiquitinated by E3 ligase NEDD4, which decreases it ability to bind EGFR. PIPKIγi5 and PI4,5P_2_ facilities SNX5 interaction with Hrs. This blocks Hrs ubiquitination by NEDD4 and is required for Hrs interaction with EGFR. Mig6 is another protein that binds to EGFR and is required for the sorting of internalized EGFR to late endosomes and the subsequently sorting to lysosomes for degradation. By interacting with NEDD4, PIPKIγi5 blocks NEDD4-mediated Mig6 ubiquitination to protect Mig6 from being degraded by proteasomes. Rab7 recruitment to early endosomes is a key step for early to late endosome maturation. PIPKIγi5 interacts with Rab7 and is required for Rab7 recruitment to early endosomes. In this way, PIPKIγi5 controls endosome maturation.

PIPKIγi5, an alternative splicing variant of PIPKIγ, was found to be localized at endosomes 12, 22, which leads to a series of research to explore the possible function of this PI4,5P_2_-producing enzyme at the endosomal system. PIPKIγi5 is required for the sorting of Epidermal Growth Factor Receptor (EGFR) from endosomes to lysosomes for degradation (**Figure 4**) 12. EGFR is a plasma membrane containing receptor tyrosine kinase that controls cell growth and differentiation during embryogenesis and adult homeostasis 77. The EGFR activation levels and the strength of its downstream signaling are tightly controlled by endocytosis and the following endosomal trafficking pathway. Upon binding with its agonists such as EGF, EGFR is activated, phosphorylated, ubiquitinated, and then quickly internalized from the plasma membrane 78. After entering the cell, EGFR is first accumulated at the limiting membrane of early endosomes, where EGFR remains activated and keeps inducing downstream signaling 78. The ubiquitinated EGFR is recognized by HGF-regulated tyrosine kinase substrate (Hrs), a component of the endosomal sorting complexes required for transport (ESCRT) 79, 80. By further recruiting other ESCRT components, Hrs mediates the initiation of early endosome limiting membrane invagination, which leads to the sorting of EGFR into intraluminal vesicles (ILVs) 81. Therefore, EGFR lost connection with its downstream effectors and this blocks EGFR-mediated signaling. Finally, ILVs-containing endosomes fuse with lysosomes and EGFR is then degraded at lysosomes. Loss of PIPKIγi5 does not affect EGFR internalization from plasma membrane, suggesting that PIPKIγi5 is not the major PI4,5P_2_-producing enzyme responsible for endocytosis. Loss of PIPKIγi5 decreases ubiquitinated EGFR interaction with Hrs, which blocks the invagination of early endosome limiting membrane and reduces EGFR sorting into ILVs. Thus, loss of PIPKIγi5 impairs the down-regulation of EGFR signaling. This function of PIPKIγi5 is mediated by its specific effector protein Sorting Nexin 5 (SNX5). SNX5 is a component of the mammalian retromer complex and is composed of a PX domain and a Bin/Amphiphysin/Rvs (BAR) domain 82. By associating with Hrs, SNX5 protects Hrs from being ubiquitinated, which can promote the Hrs interaction with EGFR. PIPKIγi5 directly interacts with SNX5, and its product PI4,5P_2_ binds to SNX5 PX domain to enhance the SNX5 binding affinity with Hrs. When PIPKIγi5 is lost, SNX5-Hrs interaction is dramatically decreased 12. Therefore, PIPKIγi5, PI4,5P_2_, SNX5, and Hrs form a signaling nexus that is required for down-regulating EGFR through the endosomal sorting pathway.

Lysosomal-associated protein transmembrane 4B (LAPTM4B) is another PIPKIγi5 effector that controls EGFR endosomal sorting 83. LAPTM4B is a four transmembrane protein mainly localized at endosomes and lysosomes 84. By binding with both Hrs and the E3 ubiquitin ligase NEDD4, LAPTM4B facilitates NEDD4-mediated ubiquitination of Hrs, which blocks Hrs interaction with ubiquitinated EGFR and inhibits EGFR sorting into ILVs. In this way, LAPTM4B impairs EGFR endosomal sorting and disrupts the down-regulation of EGFR signaling 83. The N-terminus of LAPTM4B contains a polybasic motif (PBM) with a cluster of basic arginine residues that mediates LAPTM4B binding with PI4,5P_2_. PIPKIγi5 directly interacts with LAPTM4B and the production of PI4,5P_2_ further enhances LAPTM4B-PIPKIγi5 interaction and recruits SNX5 to the same complex, which blocks Hrs interaction with LAPTM4B. Therefore, PIPKIγi5 and SNX5 antagonize the function of LAPTM4B in modulating EGFR endosomal sorting 83.

PIPKIγi5 can also regulate EGFR endosomal sorting and signaling by modulating the expression of Mitogen-inducible Gene 6 (Mig6) 85, a widely expressed tumor suppressor and adaptor protein 86, 87. Mig6 directly bind to the kinase domain of EGFR to inhibit EGFR activation 86. Furthermore, Mig6 is required for the sorting of internalized EGFR to late endosomes and the subsequently sorting to lysosomes for degradation 88. The E3 ubiquitin ligase NEDD4 mediates Mig6 ubiquitination and the subsequent degradation by proteasomes 85. By directly binding with NEDD4 and producing PI4,5P_2_, PIPKIγi5 decreases NEDD4-mediated Mig6 ubiquitination and degradation 85. Thus, PIPKIγi5 promotes the expression levels of Mig6, which contributes to the function of PIPKIγi5 in modulating EGFR endosomal sorting.

The small GTPase Ras-related protein Rab-7a (Rab7a) is a master regulator that controls the organization of endosomal and lysosomal systems 89, 90. Rab7a plays a critical role in the maturation of early endosomes into late endosomes 91. Endocytic membrane cargoes are first sorted to early endosomes, and then the early endosomes need to mature into late endosomes. Subsequently, the late endosomes fuse with lysosomes, and the endocytic cargoes are degraded by the lysosomes. Another small GTPase, Ras-related protein Rab-5a (Rab5a), is mainly located in early endosomes and plays a critical role in the organization of early endosomes 92, 93. The early to late endosome maturation requires the replacement of Rab5a by Rab7a 94-96. PIPKIγi5 directly binds with Rab7a to regulate its subcellular location and activation levels 15. PIPKIγi5 is required for the recruitment of Rab7a to Rab5a-positive early endosomes to initiate the Rab5a to Rab7a conversion. Loss of PIPKIγi5 significantly enhances Rab7a subcellular location at late endosomes and blocks Rab7a recruitment to early endosomes. This suggests that PIPKIγi5 plays fundamental role in modulating endosome maturation. A recent study reported that PI4K2A kinase mediates PI4P production at Rab7a-positive endosomes 97. PI4P is the substrate of PIPKIγi5 to produce PI4,5P_2_. The acute conversion of endosomal PI4P to PI4,5P_2_ is required for the release of Rab7a from late endosomes 97. PIPKIγi5 may be the enzyme responsible for the PI4P to PI4,5P_2_ conversion at Rab7a-positive endosomes to control Rab7a subcellular distribution.

Type II Phosphatidylinositol 4-Kinase (type II PI-4K) α and β are PI4P-producing enzymes that can localize at endosomes 98. This indicates that the PIPKIγ substrate PI4P can be synthesized at endosomes to support the production of a PI4,5P_2_ pool at endosomes. Inhibition of type II PI-4Kα kinase activity or knockdown its expression can block the EGFR sorting to late endosomes, a phenotype similar as caused by loss of PIPKIγi5 99. The endosomal PI4,5P_2_ levels must be tightly controlled and the dysregulation of endosomal PI4,5P_2_ can lead to the dysfunction of the endosomes. OCRL is a PI4,5P_2_ 5-phosphatase that downregulates PI4,5P_2_ levels at endosomes 100. Loss of OCRL results in the endosomal accumulation of PI4,5P_2_ and blocks EGFR sorting from endosome to lysosome for degradation 100. This indicates that the balance of PI4,5P_2_ levels is required for the normal endosomes function.

The endosomal network is morphologically characterized by interconnected vacuolar and tubular elements 101. The tubular structures of endosomes are thought to mediate the cargo recycling 101, 102. All three isoforms (α, β, and γ) of PIPKI can be found at endosomal tubules and they can produce PI4,5P_2_ at tubules 103. PIPKI collaborates with ACAP1 (ARF GAP with coiled-coil, ankyrin repeat, and pleckstrin homology domains 1) to modulate endosomal tubule formation. ACAP1 has a pleckstrin homology (PH) domain to bind PI4,5P_2_ and a Bin/amphiphysin/Rvs (BAR) domain to detect membrane curvature. Coexpression of PIPKI and ACAP1 can strongly induce the endosomal tubules formation 103. Sorting Nexin family proteins are also important regulators for the formation of endosomal tubular structures 102. SNX5, SNX6, and SNX9 are three members of Sorting Nexin family proteins that are PI4,5P_2_ effectors 12, 104. PI4,5P_2_ may also controls the endosomal tubule formation by modulating these three Sorting Nexins.

## Lysosomes function

Lysosome is the primary organelle responsible for the degradation of macromolecules including the extracellular materials internalized by endocytosis and intracellular components. Abnormalities in lysosomes are related to various human pathologies such as Huntington's, Parkinson's and Alzheimer's disease 72, 74, 105. PI4,5P_2_ can modulate lysosomes function by regulating the delivery of lysosomal hydrolases to lysosomes and by controlling the lysosome homeostasis 14, 15.

The catabolic capacity of lysosomes is dependent on the lysosomal hydrolases. Around 50 lysosomal hydrolases have been found at lysosomes and they are in charging of the bulk substrates degradation and pro-protein processing 106. Lysosomal hydrolases are synthesized in the endoplasmic reticulum (ER) and transported through the Golgi complex to the *trans*-Golgi network (TGN). Endosomes mediates the sorting of lysosomal hydrolases from the TGN to lysosomes. Most lysosomal hydrolases contains a mannose-6-phosphate (M6P) tag in the Golgi complex. Cation-independent Mannose 6-Phosphate Receptors (CI-MPRs) recognize this tag and target the lysosomal hydrolases to the delivery from TGN to endosomes 107. Ultimately, these hydrolases are transported to lysosomes by endosome-lysosome fusion 108, 109. CI-MPRs need to be recycled from late endosomes/lysosomes back to the TGN, which is required for CI-MPR to mediate next round of lysosomal hydrolase sorting 110. PIPKIγi5, the kinase produces PI4,5P_2_, is required for hydrolases sorting to lysosomes by modulating CI-MPR retrograde trafficking from late endosome/lysosome back to TGN. Loss of PIPKIγi5 decreases lysosomal hydrolase delivery to lysosomes and causes functional and morphological change of lysosomes 15. PIPKIγi5 loss leads to enlarged lysosomes with impaired lysosomal degradative capacity. This function of PIPKIγi5 is mediated by Rab7a and the retromer complex. Rab7a mediates the recruitment of retromer complex to late endosomes, where retromer interacts with CI-MPR to mediate the retro grade trafficking of CI-MPR from late endosomes back to TGN. By interacting with Rab7a and producing PI4,5P_2_, PIPKIγi5 promotes the Rab7a-retromer complex interaction, which is required for retromer recruitment to late endosomes. Therefore, loss of PIPKIγi5 reduces retromer recruitment to late endosomes and blocks CI-MPR recycling back to TGN. As a result, this decreases hydrolases sorting to lysosomes.

## Autophagy

**Figure 5:**
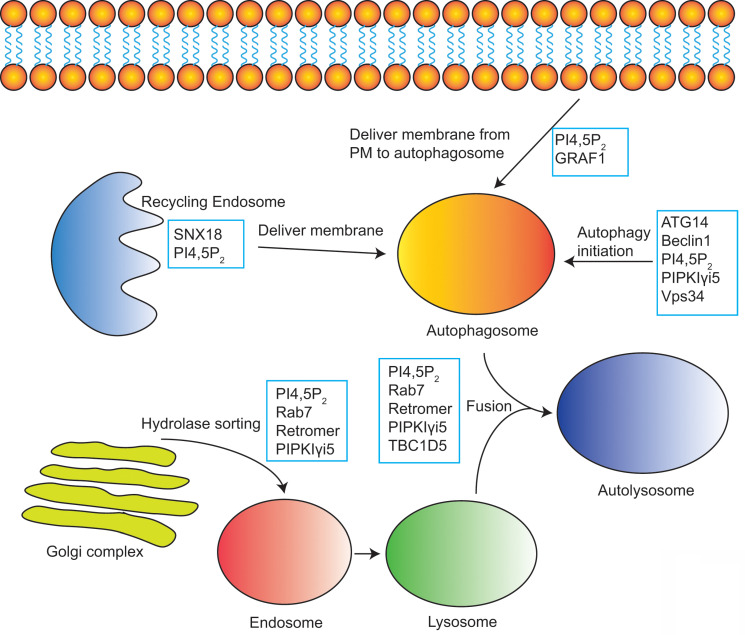
** PI4,5P_2_ in the modulation of autophagy.** PI4,5P_2_ together with its effectors and PI4,5P_2_ producing enzymes controls multiple steps of autophagy including autophagy initiation, membrane delivery from plasma membrane or recycling endosomes to autophagosome, hydrolases sorting to lysosome, and the fusion of autophagosome and lysosome to form autolysosome.

Autophagy is a self-degradative process whereby the cell digests its cytoplasmic macromolecules within lysosomes 111. At first, the cytoplasmic compartments are sequestered and engulfed by autophagosomes, then the autophagosomes fuse with lysosomes to form autolysosomes, finally the autophagy substrates are digested in autolysosomes to recycle the materials for sustaining cellular metabolism 112, 113. Deregulation of autophagy has been found in many human diseases such as cancers, vici syndrome, hereditary spastic paraparesis, lysosomal storage disorders, and Parkinson's disease 111, 114. PI4,5P_2_ and its effectors control multiple critical steps in autophagy (**Figure 5**).

PI4,5P_2_ is required for autophagosome biogenesis by controlling the internalization of plasma membrane and delivering the membrane into autophagosome precursors 115. PI4,5P_2_ is partially colocalized with phagophore proteins Atg16L1, Atg12, and Atg5 115. Depleting PI4,5P_2_ at the plasma membrane by inositol 5-phosphatase causes a block of endocytosis and a decrease of the formation of early autophagic precursors such as Atg12-positive vesicles 115, 116. PI4,5P_2_ is also required for the membrane delivery from recycling endosomes to autophagosome precursors 117. SNX18 belongs to the SNX9 family of PX-BAR proteins 118. It can be recruited to PI4,5P_2_-containing recycling endosomal membranes via binding with PI4,5P_2_ 117. By inducing membrane tabulation, SNX18 plays critical role in the delivery of membrane for phagophore expansion 117.

PIPKIγi5, an endosomal localized PI4,5P_2_ producing enzyme, plays important roles in both autophagy initiation and the final autophagy substrates degradation. PIPKIγi5 is required for normal autophagic degradation by modulating the lysosomes function 15. Loss of PIPKIγi5 leads to autolysosome dysfunction and blocks the autophagy substrates digestion at autolysosomes. Furthermore, it is also reported that PIPKIγi5 and PI4,5P_2_ can modulate the initiation process of autophagy 13. The autophagy-related protein 14 (ATG14) and VPS34 (a class III phosphatidylinositol 3-kinase) play key function in the initiation of autophagy. ATG14 contains a C-terminal Barkor/ATG14(L) autophagosome-targeting sequence (BATS) domain that binds with PI4,5P_2_, which stabilizes the ATG14 interaction with VPS34 and Beclin1. This assembly of ATG14-VPS34-Beclin1 complex is critical for the autophagy initiation. By interacting with ATG14 and producing PI4,5P_2_, PIPKIγi5 facilitates the ATG14 complex formation. Loss of PIPKIγi5 causes a loss of ATG14 and Beclin1, which blocks the autophagy initiation 13.

PI4,5P_2_ also modulates the autophagosome/lysosome fusion. Phosphatidylinositol 4-kinase type IIα (PI4KIIα) produces PI4P at lysosomes, which is required for autophagosome/lysosome fusion 119, 120. The conversion of PI4P to PI4,5P_2_ at lysosomes inactivates Rab7a to mediate the release of Rab7a effector PLEKHM1 from lysosomes, which is a critical step for the fusion of lysosome with autophagosome 121. PIPKIγi5 may be an important enzyme responsible for the PI4P to PI4,5P_2_ conversion and Rab7a inactivation at lysosomes. PIPKIγi5 can localize to lysosomes. By producing PI4,5P_2_ and direct interacting with Rab7a, PIPKIγi5 promotes the Rab7a-retromer interaction at lysosomes 15. Retromer further recruits TBC1D5, the Rab7a‐specific GTPase‐activating protein (GAP), to lysosomes 15, 122. Then TBC1D5 mediates the conversion of active Rab7a (GTP-bound) to inactive Rab7a (GDP-bound).

Autophagic lysosome reformation (ALR) is the process whereby nascent lysosomes are formed from membranes of autolysosomes. ALR is critical for the lysosome homeostasis maintenance. During ALR, tubular structures extrude from autolysosomes, and then small proto-lysosomes pinch off from the reformation tubules. Proto-lysosomes are finally maturated to new functional lysosomes 123. PI4,5P_2_ and clathrin play critical roles in ALR 14. PIPKIβ, a kinase producing PI4,5P_2_, can be recruited to autolysosomes and convert autolysosome-localized PI4P to PI4,5P_2_, which causes the recruitment of clathrin adaptor AP2 14. Consequently, AP2 recruits clathrin to autolysosomes and induce the membrane curvature and initiation of membrane budding. These processes lead to the reformation tubule extrusion. PIPKIα, another kinase responsible for PI4,5P_2_ production, is found to localized at the reformation tubules. By generating PI4,5P_2_, PIPKIα recruits clathrin to reformation tubules, which is required for the pinching off of proto-lysosomes 14.

## Exocytosis

**Figure 6:**
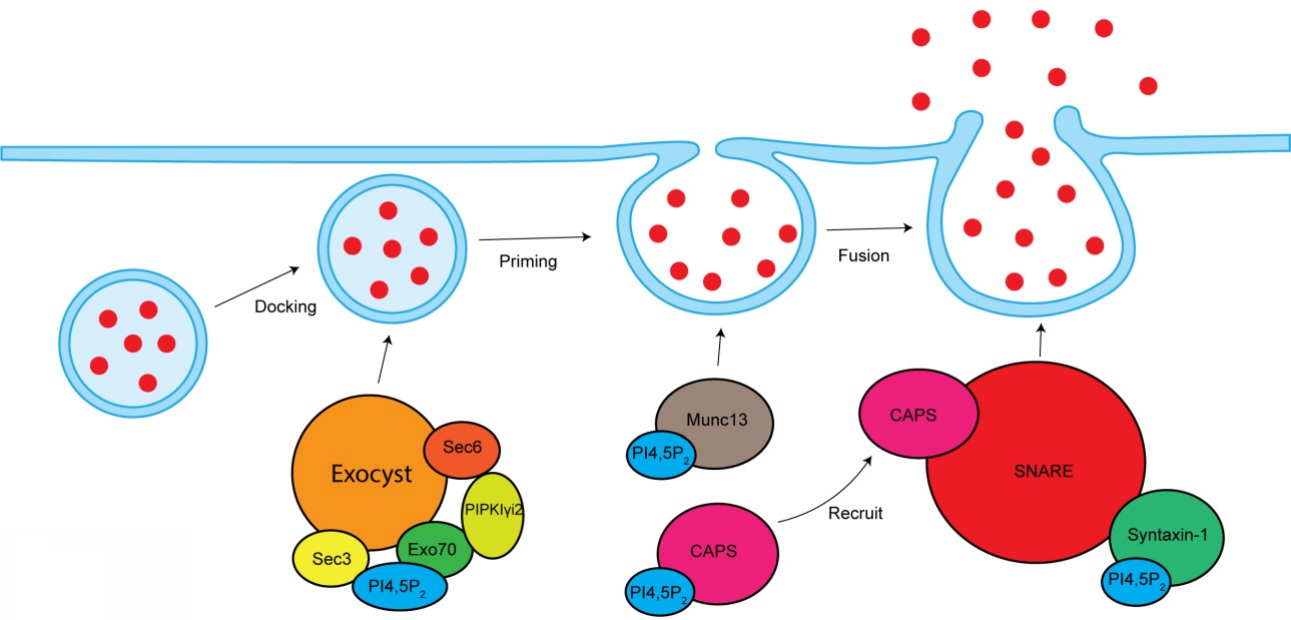
** PI4,5P_2_ in the modulation of exocytosis.** PI4,5P_2_ is required for exocyst-mediated secretory vesicles sorting to plasma membrane. By interacting and modulating Munc13 and CAPS, PI4,5P_2_ modulates vesicle priming. By modulating SNARE complex, PI4,5P_2_ controls vesicles fusion with plasma membrane.

Exocytosis is a fundamental membrane trafficking event that release intracellular protein contents such as neurotransmitters, hormones, and cytokines to the cell exterior 124, 125. It also mediates the polarized delivery of proteins and lipids to specific domains of the plasma membrane 126. During exocytosis, secretory vesicles undergo different trafficking steps: docking process for recruiting and tethering the vesicles to the plasma membrane, priming process for vesicles maturation to attain fusion-competence, fusion process to fuse with plasma membrane and finally release the vesicle contents 125. PI4,5P_2_ and its effector play important roles in all these steps of exocytosis (**Figure 6**).

Vesicle exocytosis occurs at PI4,5P_2_-rich membrane domains 127. Locally depleting PI4,5P_2_ at vesicle docking sites leads to significantly vesicle undocking from the plasma membrane 128. Knockout of PIPKIγ in mouse causes synaptic transmission defects, a smaller readily releasable pool of synaptic vesicles, and a delay in fusion pore expansion 45, 129.

CAPS and Munc13 are two PI4,5P_2_ effector proteins that are critical for the vesicle priming. PI4,5P_2_ binds to the PH domain of CAPS, which is required for CAPS activation to stimulate fusion 130, 131. PI4,5P_2_ present is also required for CAPS to recruit the formation of SNARE complex, a key step to initiate vesicle fusion 132. The C2B domain of Munc13 binds to PI4,5P_2_ in a Ca^2+^-dependent manner 133. Munc13 is cytoplasmic, but Ca^2+^ can stimulate its translocation to PI4,5P_2_-rich plasma membrane domains, which is required for its activity 131. SNARE proteins are play critical role in the vesicle fusion 134. PI4,5P_2_ interacts with SNARE proteins and modulates SNARE function. Syntaxin-1, a key component of SNARE proteins, binds to PI4,5P_2_, which modulates syntaxin-1 clustering 135. Depleting PI4,5P_2_ at cell membranes by the 5-phosphatase synaptojanin-1 eliminated syntaxin-1 clusters 135, which further supports the function of PI4,5P_2_ in modulating syntaxin-1 and SNARE proteins. Synaptotagmin is a tandem C2 domain-containing protein that can bind PI4,5P2 136. Synaptotagmin can facilitate the inducing of membrane curvature to promote fusion process 136, 137.

The exocyst is an octameric protein complex that mediates the polarized tethering of secretory vesicle to the plasma membrane before the exocytic fusion 138. The interaction between exocyst and PI4,5P_2_ is required for exocyst to target the secretory vesicles to plasma membrane. Sec3 and Exo70, two subunits of exocyst, directly interacts with PI4,5P_2_ via their conserved basic residues 139. The Exo70-PI4,5P_2_ interaction is required for the docking and fusion of post-Golgi secretory vesicles 139. Exo70 mutant with deficiency to bind PI4,5P_2_ lost the ability to recruit other exocyst components to the plasma membrane 139. These reports indicate important role of PI4,5P_2_ in mediating exocyst function. PIPKIγi2, a kinase producing PI4,5P_2_, directly binds to exocyst subunits sec6 and Exo70 24. PIPKIγi2 also interacts with talin 140, a critical protein controlling adhesion turnover. By coordinating exocyst and talin, PIPKIγi2 facilitates the polarized sorting of integrin to plasma membrane 24.

Exosome is a specific type of secreted extracellular vesicle with the size about 30-200 nm in diameter 141. Exosomes can mediate cellular information exchange by transferring RNA, proteins, and lipids contained inside of the exosomes 142. In this way, exosomes play critical roles in many biological processes including antigen presentation, cellular homeostasis, angiogenesis, and apoptosis 142. Exosomes are generated from the multivesicular body (MVB, or late endosomes), which are formed by inward budding of the limited membrane of endosomes to produce small intracellular vesicles (ILVs) 143. ILVs are the precursors of exosomes. The MVB can be sorted towards to plasma membrane, then the fusion with plasma membrane releases ILVs to the extracellular fluid 144. The released ILVs are so called exosomes. Although very less knowledge is currently known about the PI4,5P_2_ function in exosomes, some reports suggest that PI4,5P_2_ may play important roles in exosome production and function. EGFR-containing exosomes have been widely found in cancer microenvironments and can promote cancer metastasis 145, 146. PIPKIγi5 is localized at endosomes and the production of PI4,5P_2_ by PIPKIγi5 is required for ESCRT complex-mediated EGFR invagination from the endosome limiting membrane to form the ILVs 12. Therefore, PI4,5P_2_ pathway may affect the packaging of EGFR into the exosome precursors. ARF6 is a critical regulator of exosomes by controlling the budding of endosomes to form ILVs 147. ARF6 is highly involved in PI4,5P_2_ pathway by interacting and activating the PI4,5P_2_-producing kinase PIPK 148. It is possible that ARF6 can modulate PI4,5P_2_ production at endosomes to regulate ILVs budding. More research is required to further determine the function of PI4,5P_2_ pathway in exosome formation and function.

## PI4,5P_2_ pathway dysregulation and disease

Membrane trafficking dysfunction is highly related to the development of many diseases. As PI4,5P_2_ pathway plays critical roles in membrane trafficking, the dysregulation of PI4,5P_2_ pathway has been found in diseases such as Lowe syndrome, neuronal disorders and cancer.

Defective of the gene *OCRL* causes Lowe oculocerebrorenal syndrome, which is characterized by congenital cataracts, central hypotonia, mental retardation, and proximal renal tubular dysfunction 149. OCRL is an inositol polyphosphate 5-phosphatase that hydrolyzes the 5-phosphate of PI4,5P_2_ and converts PI4,5P_2_ to PI4P 149. OCRL is localized at endosomes and controls endosomes function. Loss of OCRL leads to abnormal accumulation of PI4,5P_2_ at endosomes and causes defects of multiple membrane receptors recycling 100. OCRL can also directly bind to clathrin and loss of OCRL leads to endocytic defects 150. The function of OCRL suggests that the dysregulation of PI4,5P_2_ levels at endosomes or clathrin-coated vesicles may contribute to the pathological process of Lowe syndrome.

PI4,5P_2_ pathway dysregulation is reported in neuronal disorders. PI4,5P_2_-producing kinase PIPKIγ is highly expressed at neuronal system. Knockout of pan-PIPKIγ in mice leads to the synaptic defects and causes the postnatal lethality 45. The PI4,5P_2_ 5-phosphatase Synaptojanin 1 also plays critical roles in the neuronal system. In Synaptojanin 1-knockout mice, PI4,5P_2_ levels and clustering of clathrin-coated vesicles are both enhanced in the neurons 47. PI4,5P_2_ may also have a correlation with the development of Alzheimer's disease as the PI4,5P_2_ levels are decreased in the Alzheimer-diseased brains 151. The possible role of PI4,5P_2_ pathway in Alzheimer's disease still need to be clarified.

By interacting with distinct effectors and localized at specific subcellular locations, different PI4,5P_2_-producing kinases may play diverse function in cancer. For example, PIPKIγi2 is localized to cell adhesions by interacting with talin. By modulating talin assembly to adhesions and mediating β1-integin recycling endosomes trafficking to plasma membrane, PIPKIγi2 promotes cancer cell directional migration and invasion 24, 152. Therefore, loss of PIPKIγi2 decreases breast cancer migration and invasion 153. Consistently, decreased PIPKIγi2 expression correlates with better breast cancer patients' prognosis 153. PIPKIγi5, another splice variant of PIPKIγ, is localized at endosomes. PIPKIγi5 interacts with the oncogene LAPTM4B. Expression of LAPTM4B is elevated in many types of cancers and serves as a positive indicator for poor prognosis 154. LAPTM4B blocks EGFR sorting to lysosome for degradation and thereby promotes EGFR signaling. PIPKIγi5 neutralizes the function of LAPTM4B by blocking EGFR up-regulation 83. Furthermore, PIPKIγi5 is critical for the stable expression of Mig6, a tumor suppressor that directly binds to EGFR and inhibits EGFR activation 155. Therefore, PIPKIγi5 down-regulates EGFR signaling and may function as a tumor suppressor.

## Conclusions

PI4,5P_2_ plays critical roles in multiple membrane trafficking events happening at diverse subcellular locations including the plasma membrane, endosome, Golgi system, lysosome, autophagosome, and autolysosome. The multifunction of PI4,5P_2_ is dependent on the diversity of PI4,5P_2_-producing enzymes and the PI4,5P_2_ effector proteins. Different PIPKs mediate the local production of PI4,5P_2_ at distinct subcellular locations, and then specific PI4,5P_2_ effectors are recruited to organize the membrane trafficking events.
